# The parathyroid glands identification of carbon nanoparticles via preoperative injection in reoperation of recurrent benign multinodular goiter

**DOI:** 10.3389/fendo.2024.1361736

**Published:** 2024-11-26

**Authors:** Yonghui Wang, Quancai Li, Mingxiu Fan, Kunxiu Ming

**Affiliations:** ^1^ Department of Thyroid and Breast Surgery, Weifang People’s Hospital, Weifang, Shandong, China; ^2^ Department of Neurosurgery, Weifang People’s Hospital, Weifang, Shandong, China; ^3^ Department of Central Sterile Supply, Weifang People’s Hospital, Weifang, Shandong, China

**Keywords:** reoperation, thyroid, benign multinodular goiter, carbon nanoparticles, hypoparathyroidism

## Abstract

**Introduction:**

Benign multinodular goiter (BMNG) can grow very large and cause compression symptoms, making the operation procedure difficult. However, the recurrence rate of BMNG ranges from 3% to 43%. Reoperative thyroid surgery for BMNG is uncommon and can result in a high rate of complications, including hypoparathyroidism and recurrent laryngeal nerve palsy. Carbon nanoparticles (CNs) have been widely used as a protective agent for the parathyroid gland and as a tracer agent in central lymph node dissection. However, the protection effect of CNs in redoing BMNG has not been well illustrated. This study investigates whether CNs could protect parathyroid glands (PGs) during reoperation for patients with BMNG.

**Methods:**

BMNG patients who previously underwent thyroidectomy and received reoperation between January 2019 and January 2022 were retrospectively recruited. The Dunhill approach was employed for all patients. The patients were divided into two groups: the CNs group, who received injection CNs injection 1 hour before the operation (n = 24), and the control group, who underwent thyroid surgery without CNs injection (control group, n = 25). The numbers of PGs preserved *in situ*, autotransplantation, the accidental removal of the PGs, and the parathyroid hormone level were recorded and analyzed.

**Results:**

The results revealed that more PGs were preserved *in situ* in the CNs group compared to the control group (3.25±0.15 *vs* 2.60±0.16, P=.007). Moreover, fewer PGs were subjected to autotransplantation and were accidentally discovered in the specimen in the CNs group compared to the control group. Patients who had CNs injection exhibited a lower rate of transient (5/24 *vs.* 13/25, P=.024) and permanent hypoparathyroidism (2/24 *vs.* 9/25, P=.020) compared to the control group.

## Introduction

Benign multinodular goiter (BMNG) is the most popular endocrine disease, especially in the iodine-deficiency areas (1). Generally, BMNG is not treated if the patient has normal thyroid function and no compressive symptoms (2). Surgery for BMNG is necessary for those with compressive symptoms, large substernal goiter, suspected malignancy, and cosmetic concerns (2). The extent of resection for BMNG has varied over the years but focused on removing all involved diseases (1). However, the surgery complication rate has significantly increased with the extent of resection (1). L-thyroxin therapy has been advocated to decrease the recurrence rate but with minimal efficiency (3, 4). The recurrence rate of BMNG ranges from 3% to 43% (5–7).

Reoperative thyroid surgery has dramatically increased the incidence of complications, particularly hypoparathyroidism and recurrent laryngeal nerve (RLN) palsy (8). The intraoperation monitoring of RLN has significantly reduced RLN injury (9, 10). Fine dissection operation to protect the parathyroid in the thyroid operation has been recommended (8, 11). However, scar formation, landmark distortion, and tissue friability complicated efforts to identify and protect the parathyroid (8). Carbon nanoparticles (CNs) as lymph node tracers have been widely used in stomach, breast, and thyroid cancers (12–14). Given that the space between capillary endothelial cells is between 20 and 50 nm, smaller than the CN diameter (150 nm), CNs could not transport via the capillary. Therefore, the thyroid and lymph node were stained black, while the parathyroid was still in its original color if CNs were injected into the thyroid (15). Recent studies have shown that applying CNs in thyroid surgery has significantly decreased the rate of hypoparathyroidism and increased the number of dissection lymph nodes (15). However, the leakage of CNs may drain the tissue, including the parathyroid around the thyroid, making identifying the parathyroid more difficult (15). The purpose of this study was to investigate whether CNs via preoperative injection could identify and protect the parathyroid during reoperation for recurrent BMNG.

## Materials and methods

### Patients

From January 2019 to January 2022, 49 consecutive patients who performed reoperative thyroid surgery at the Department of Thyroid and Breast Surgery, Weifang People Hospital, were retrospectively enrolled. Patients were divided into the CNs injection group (CNs group, n=24) and the control group (n=25).

Inclusion criterion: Patient have a history of thyroid surgery, with pathology indicating BMNG. The patient presents compression symptoms and a tumor whose longest diameter exceeds 40 mm with postoperative pathology confirmation of BMNG.

Exclusion criterion: The patient has a history of neck radiotherapy. The patient also has RLN palsy or hypoparathyroidism before the secondary thyroid surgery with postoperative pathology confirmation of a malignant tumor.

All the redoing thyroid surgery was performed by the same professional thyroid surgeon (Yonghui Wang).

### CNs injection before the operation

**Figure 1:**
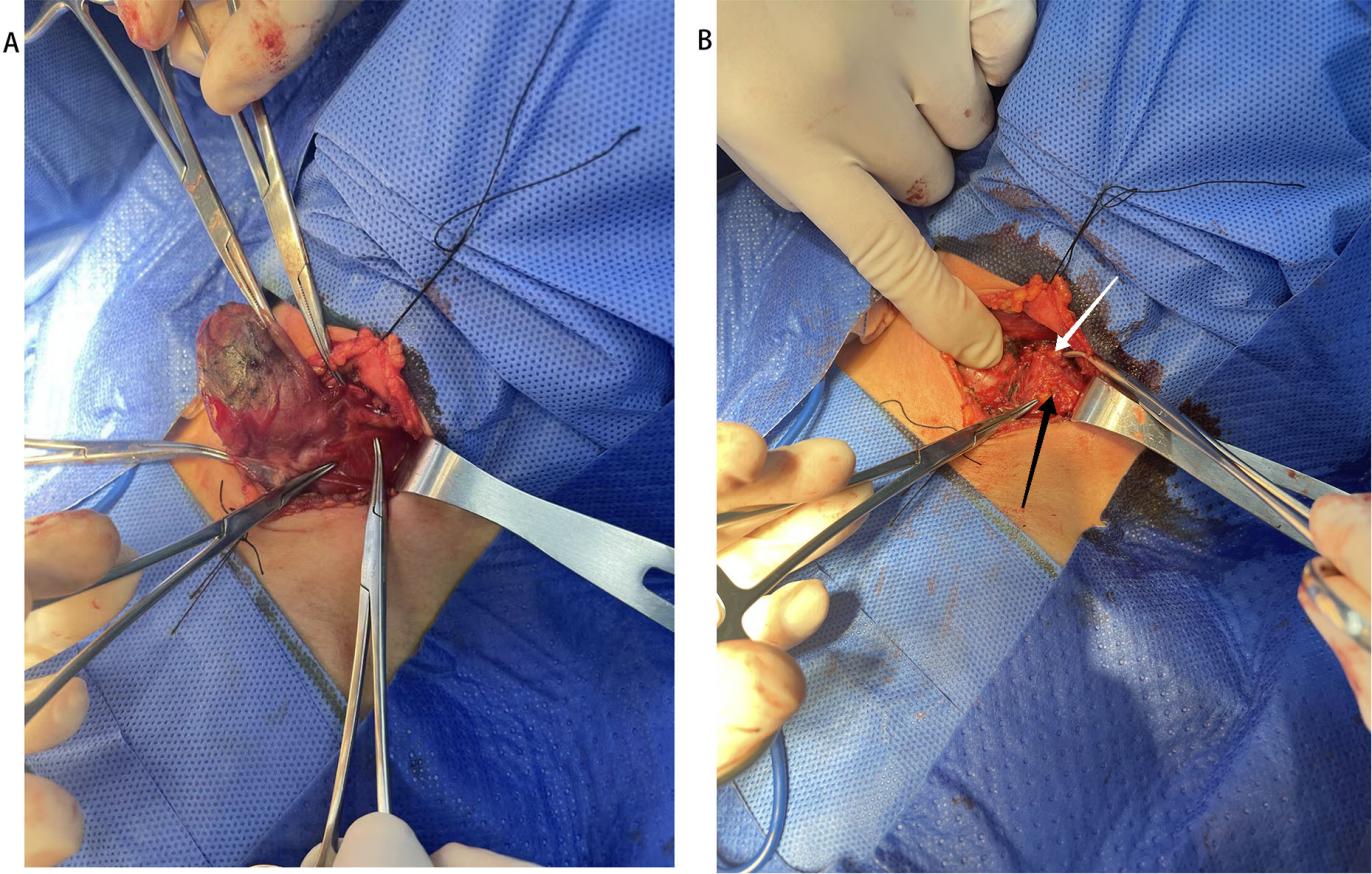
Injection of Carbon nanoparticles. **(A)** Preparation of Carbon nanoparticles. **(B)** The Carbon nanoparticles injection under the guiding of ultrasound.

Ultrasound-guided injection of CNs was performed 1 hour before the surgery in the CNs group. Carbon nanoparticles (0.5 mL per ampoule, Chongqing LaiMei Pharmaceutical Co., Ltd., Chongqing, China) were used. The special procedures were as follows: 0.6 mL of CNs was extracted with a 1 mL syringe, and the air in the needle was expelled (**Figure 1A**). Under the ultrasound guide, CNs were slowly injected into the gland at the superior, middle, and inferior parts of every thyroid lobe (0.1 mL at each point). The needle should be gently withdrawn with negative pressure after injection (**Figure 1B**) and immediately pressed for 3 min with gauze. To avoid the skin staining by CNs, we firstly changed a new needle, then expelled the air from the needle as much as possible. In addition, after the injection, the needle gently withdrawn with negative pressure. Preoperative injection of CNs was performed by an experienced surgeon (Yonghui Wang) 1 hour before the operation.

### Surgical procedure

All the patients underwent the Dunhill approach (hemithyroidectomy with counterlateral subtotal resection). After general anesthesia with tracheal intubation was administered, patients were placed supine with cervical hyperextension. The reoperation approach was through the scar of the previous Kocher’s incision, with the linea alba cervicalis split, exposing the thyroid. The isthmus of thyroid was break along the trachea. Then total right lobectomy and subtotal left lobectomy were performed. The first step was ligation of the inferior thyroid vessels. Then, the RLN was identified in the tracheoesophageal groove and traced very carefully along its cervical course. Thirdly, the superior thyroid vessels were ligated. At last, the right thyroid lobe was total removed. While for the left lobe, the superior pole of the thyroid turned up and discovered the superior parathyroid gland, then preserved the back of the superior pole of thyroid and removed the most of the left thyroid lobe. A nerve stimulator was used to monitor the effect of RLN in all cases. All the patients were performed indirect laryngoscopy three days post-surgery. If RLN palsy was observed, indirect laryngoscopy was performed 6 months post-surgery to confirm persistent RLN palsy, indicated by the vocal cord remaining in the middle position.

### Monitoring indicators

Parathyroid (PG) related parameters (number of PGs preserved *in situ*), number of PGs autotransplantation, number of PG accidentally removed, and postoperative parathyroid hormone level (PTH) were collected and analyzed.

The level of PTH would be tested at three time points: preoperation, 1 day, and 3 months after the operation. Hypoparathyroidism is defined as a decline in serum PTH below 15 pg/mL. The patient was considered to have permanent hypoparathyroidism when the serum PTH level at 3 months after surgery was below 15 pg/mL.

### Statistical analysis

Continuous variables were presented as mean ± standard deviation and compared using independent samples t-tests. Chi-square tests were performed to analyze categorical data. Statistical analysis was performed by SPSS 17.0, and p < 0.05 was considered statistically significant.

## Results

### Patients’ characteristics

**Figure 2:**
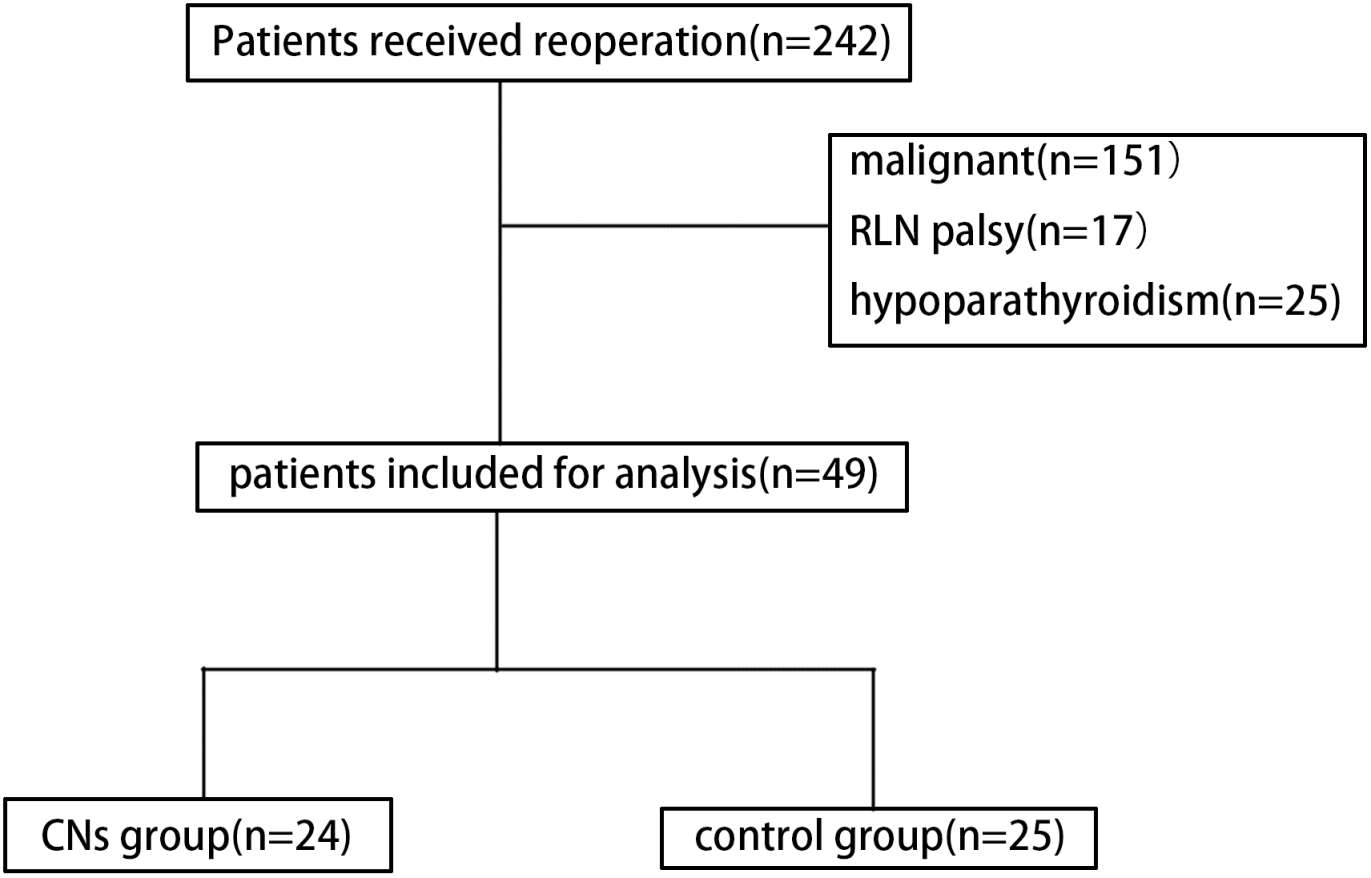
Diagram showing the patient inclusion and exclusion criteria. (CNs, carbon nanoparticles).

The process of patient selection based on the inclusion and exclusion criteria is shown in **Figure 2**. No death was noted in both groups, and no toxic side effect due to CNs was observed in the CNs group. As summarized in **Table 1**, no significant difference was noted in clinical characteristics, including age, sex, and Hashimoto’s thyroiditis, between the two groups. No significant difference was observed between them regarding the first surgical approach, the PTH level before the reoperation, the interval between the first surgery and reoperation, and the nodule size.

**Table 1:** The clinical characteristics of the patients in the CNs group and control group.

characteristics	CNs group	control group	P value
Age	59.63±7.87	61.12±4.67	0.426
Sex			0.950
Male	20	21	
Female	4	4	
Hashimoto’s thyroiditis			0.138
Yes	2	6	
No	22	19	
First surgical approach			0.588
Partial hemithyroidectomy	18	17	
Bilateral partial hemithyroidectomy	6	8	
Interval (Y)	34.25±9.42	38.92±10.30	0.105
nodule size (cm)			0.897
< 5 cm	13	14	
≥5 cm	11	11	

CNs, Carbon nanoparticles.

### PG identification and protection

**Figure 3:**
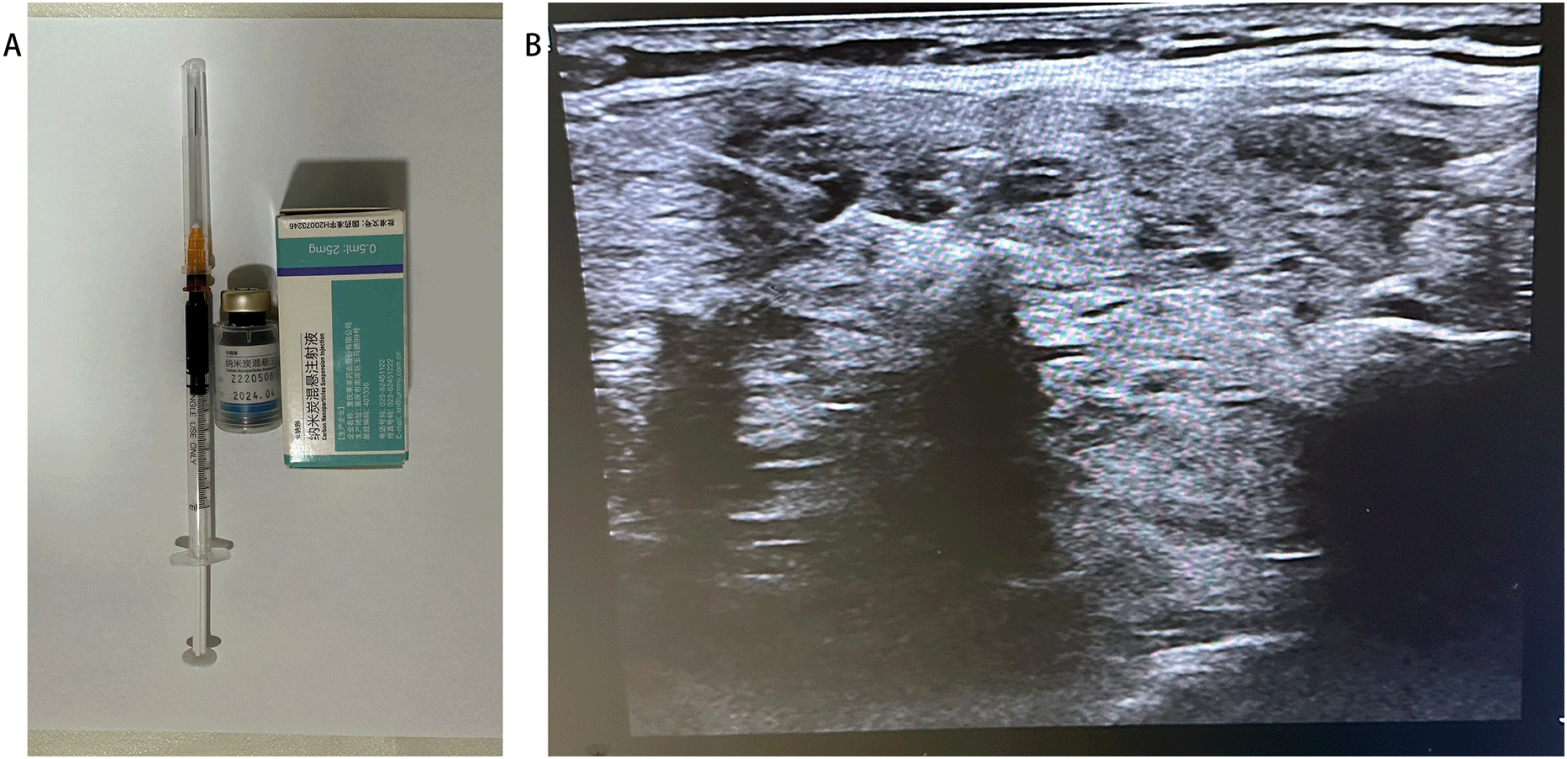
**(A)** residual thyroid gland (black arrow). **(B)**. left superior (black arrow) and inferior (white arrow) parathyroid gland (white arrow).

During the operation, the residual thyroid gland was stained black, whereas the PGs and surrounding adipose tissues were not (**Figures 3A, B**). **Table 2** shows that more PGs in the CNs group were preserved *in situ* compared to the control group. Out of the studied cases, four patients in the CNs group and eleven cases in the control group underwent PGs autotransplantation. The difference between the two groups was statistically significant. Three cases in the CNs group and fourteen cases in the control group had PGs detected in the specimen after the surgery, with a significant difference between the two groups. Twenty-four hours after the surgery, the PTH level in 5 patients of the CNs group and 13 patients of the control group was lower than the normal state, with a statistical difference between the two groups (**Table 3**). Six months after the surgery, two patients in the CNs group and nine patients of the control group were diagnosed with persistent hypoparathyroidism.

**Table 2:** PGs preserved in situ, PGs autotransplanted, PGs accidentally discovered in the CNs group and control group.

	CNs group	control group	P value
PGs preserved in situ	3.25±0.15	2.60±0.16	0.007
PGs autotransplanted			0.038
No	20	14	
Yes	4	11	
PGs accidentally discovered			0.001
No	21	11	
Yes	3	14	

PG, parathyroid; CNs, Carbon nanoparticles.

**Table 3:** Hypoparathyroidism after the surgery.

	CNs group	control group	P value
Transient hypoparathyroidism			0.024
No	5	12	
Yes	19	13	
Persistent hypoparathyroidism			0.020
No	2	9	
Yes	22	16	

CNs, Carbon nanoparticles.

### Postoperative complications

No postoperative deaths were reported. The other complications of thyroid reoperation were RLN injury, bleeding, and wound infection (**Table 4**). All the patients completed 6 months of follow-up. Transient RLN injury was found in two patients with two RLNs in the CNs group and three patients with three RLNs in the control group. No statistically significant difference was found between the two groups. After 6 months, the function of RLN of the five patients returned to normal. Three patients (two in CNs group and one in the control group) had reoperation for the bleeding. Three patients (one in CNs group and two in the control group) had wound infection. Both bleeding and wound infection occurrences in the two groups have no significance.

**Table 4:** The other postoperative complications.

	CNs group	control group	P value
RLN injury			
Transient	2	3	1.000
Permanent	–	–	–
Postoperative bleeding	2	1	0.609
Would infection	1	2	1.000

RLN, recurrent laryngeal nerve; CNs, Carbon nanoparticles.

## Discussion

Scar formation, landmark distortion, and tissue friability made reoperative thyroid surgery difficult (8). Thus, advanced experience and skills are required to perform reoperative thyroid surgery. However, reoperative thyroid surgery remains a dangerous procedure because of the relatively high rates of complication including permanent RLN palsy and hypoparathyroidism compared to primary thyroid surgery (8). Both complications can decrease the quality of patients’ life. Therefore, protecting RLN and parathyroid is important (16). CNs were used to identify and protect the parathyroid during the reoperative BMNG procedure.

BMNG is the most common endocrine disease requiring surgery (1). In our series, the most common benign reoperative thyroid surgery is BMNG. Two main reasons for the recurrence of BMNG are as follows: First, the hospital is located in an iodine-deficiency region with high rates of multinodular goiter and autoimmune thyroiditis. Second, most patients with thyroid disease underwent partial thyroidectomy during the initial surgery due to surgeons with limited thyroid surgery experience. For the reoperation of BMNG, three popular surgical approaches were included in our choice: the Dunhill procedure, total thyroidectomy, and bilateral subtotal resection (1). Recurrence was observed in 0.5% of patients treated with total thyroidectomy, 5% were treated with the Dunhill procedure, and 12% underwent bilateral subtotal resection at the 5-year follow-up after the surgery (1). In our series, we mainly chose the Dunhill procedure because of the relatively low recurrence rate compared to the bilateral subtotal resection and low complications compared to the total thyroidectomy.

Hypoparathyroidism is one of the major complications of reoperation (17). Hypocalcemia induced by hypoparathyroidism could lead to a series of problems (17). The incidence of transient hypoparathyroidism is 10.9% for total thyroidectomy and 4.2% and 2.1% for Dunhill procedure and subtotal bilateral resection, respectively, during primary thyroid surgery (18–20). However, the incidence of transient hypoparathyroidism after reoperation is 0% to 47.3%, while that of permanent hypoparathyroidism is 0% to 7.6% (21, 22). Temporary hypoparathyroidism manifested in fingers and toes numbness. Hypocalcemic tetany might happen in some severe cases. These symptoms immediately disappear after intravenous or oral administration of calcium. The features of permanent hypoparathyroidism include temporary hypoparathyroidism and induced anxiety and depression. Therefore, these patients need long-term oral supplements of calcium. However, the additional calcium supplement could induce bone and joint injury. Therefore, permanent hypoparathyroidism needs more medical therapy to maintain physical and psychological health. The extent of resection, surgical technique, and recurrent goiter were the risk factors for hypoparathyroidism in thyroid surgery. Concerning the protection of parathyroid, we used a relatively lower complication approach of the Dunhill procedure (21, 22). Several strategies have been applied to visualize PGs intraoperatively. The fine dissection operation to protect the parathyroid in the thyroid operation has been advocated recently. However, the parathyroid glands are often difficult to identify because of the parathyroid ectopia or sanguine in the operative field. Meanwhile, identifying the parathyroid for the reoperation thyroid is challenging due to the adhesion and scar formation in the operative area, which dramatically changed the anatomical structure. Nuclide was used as a radiotracer for intraoperative localization of adenomatous PGs via gamma probe identification. However, it has not been widely used because of the special expensive equipment and radioactive contamination (23, 24). Methylene blue is the most widely used staining agent, which has the advantage of identifying enlarged PGs such as parathyroidoma. However, it can also falsely stain the thyroid, lymph nodes, and normal PGs (25). Recent studies have shown that applying CNs in thyroid surgery has significantly decreased the rate of hypoparathyroidism and increased the number of dissection lymph nodes. However, the leakage of CNs might contaminate the tissue, including the parathyroid around the thyroid, which made identifying the parathyroid more difficult. Moreover, the effect of CNs in the reoperative BMNG has not been well illustrated. This study has applied the preoperative injection of CNs to avoid leakage and identify the parathyroid. Through applying CNs and protective measures for the parathyroid in the reopreative BMNG, we have gained valuable clinical experience. (a) Administering under ultrasound guidance, a 1 ml syringe was selected for the injection before the surgery. (b) Injection was administered at three points (superior, immediate, and inferior poles) with 0.1 ml of CNs injection at each point. (c) The tip of the syringe was located in the middle of normal thyroid tissue. (d) The tip of the syringe in the vessel was avoided by withdrawal before injection. (e) Negative pressure should be adopted to withdraw the needles, and the injection sites should be immediately pressed for 3 min with the gauze. However, in this series, with the assistance of the negative tracing, most of the parathyroid have been discovered, persistent hypoparathyroidism has been happened for 2 patients. The main reason might be that the capillaries around the parathyroid have been destroyed if the parathyroid was discovered. Therefore, in order to protect the capillaries around the parathyroid, we have selected the Dunhill procedure although the recurrence of total thyroidectomy was lower compared to the Dunhill procedure.

Another dangerous complication of thyroid surgery is RLN injury. The incidence of transient RLN palsy for total thyroidectomy was 5.5% higher compared to 4% and 2% for the Dunhill procedure and subtotal bilateral resections, respectively (18–20). The risk factors of RLN injury included the surgery approach, the surgeon’s experience, and the type of surgery (reoperation or primary surgery) (18–20). Intraoperative monitoring of RLN has been used in thyroid surgery and significantly decreased RLN injury (9, 10). In this study, transient RLN palsy occurred in five patients with no permanent RLN injury.

This study has some limitations. First, it is a retrospective study with a relatively limited number of cases, thus carrying the possibility of selection bias. Second, the number of parathyroid before the surgery could not be confirmed, potentially impacting the conclusion of this study. Future prospective studies or randomized controlled trials should be performed to ensure the protection effect of CNs’ reoperative thyroid surgery.

The Dunhill procedure seems to be a good choice for the reoperation BMNG. The application of CNs via preoperative injection in reoperation BMNG could significantly decrease the occurrence of transient and permanent hypoparathyroidism.

## Data Availability

The original contributions presented in the study are included in the article/supplementary material. Further inquiries can be directed to the corresponding author.
